# Awareness, incidence and clinical significance of acute kidney injury after non-general anesthesia: A retrospective cohort study: Erratum

**DOI:** 10.1097/MD.0000000000012628

**Published:** 2018-09-28

In the article, “Awareness, incidence and clinical significance of acute kidney injury after non-general anesthesia: A retrospective cohort study”,^[1]^ which appeared in Volume 97, Issue 35 of *Medicine*, the authors would like to add the following funding information: This research was supported by a grant from the KoreaHealth Technology R&D Project through the Korea Health Industry Development Institute (KHIDI), funded by the Ministry of Health & Welfare, Republic of Korea (grant number: HI17C1827).

Reference 20 should be Park S, Baek SH, Ahn S, et al. Impact of electronic acute kidney injury (AKI) alerts with automated nephrologist consultation on detection and severity of AKI: a quality improvement study. *Am j Kidney Dis* 2017.
